# An overview of implementing an evidence based program to increase HPV vaccination in HIV community clinics

**DOI:** 10.1186/s12889-022-14100-0

**Published:** 2022-09-07

**Authors:** Jessica Wells, James L. Klosky, Yuan Liu, Theresa Wicklin Gillespie

**Affiliations:** 1 Nell Hodgson Woodruff School of Nursing, Emory University, 1520 Clifton Road, NE, RM. 230, Atlanta, GA 30324 USA; 2 Department of Pediatrics, School of Medicine, Emory University, Atlanta, GA USA; 3 Aflac Cancer and Blood Disorders Center, Children’s Healthcare of Atlanta, Atlanta, GA USA; 4 Departments of Biostatistics and Bioinformatics, Emory University, Atlanta, GA USA; 5 Winship Cancer Institute, Emory University, Atlanta, GA USA; 6 Department of Surgery, Division of Surgical Oncology, School of Medicine, Emory University, Atlanta, GA USA

**Keywords:** HIV, HPV vaccination, Implementation

## Abstract

**Background:**

HPV-related anal cancer occurs in excess rates among people living with HIV (PLWH) and has been increasing in incidence. The HPV vaccine is an effective and safe approach to prevent and reduce the risk of HPV-related disease. Yet, HPV vaccine programs tailored and implemented in the HIV population are lagging for this high-risk group.

**Methods:**

A pre-post intervention study design will be used to tailor, refine, and implement the 4 Pillars™ Practice Transformation Program to increase HPV vaccination among PLWH. Guided by the RE-AIM framework, the CHAMPS study will provide training and motivation to HIV providers and clinic staff to recommend and administer the HPV vaccination within three HIV clinics in Georgia. We plan to enroll 365 HIV participants to receive HPV education, resources, and reminders for HPV vaccination. Sociodemographic, HPV knowledge, and vaccine hesitancy will be assessed as mediators and moderators for HPV vaccination. The primary outcome will be measured as an increase in uptake rate in initiation of the HPV vaccine and vaccine completion (secondary outcome) compared to historical baseline vaccination rate (control).

**Discussion:**

The proposed study is a novel approach to address a serious and preventable public health problem by using an efficacious, evidence-based intervention on a new target population. The findings are anticipated to have a significant impact in the field of improving cancer outcomes in a high-risk and aging HIV population.

**Trial registration:**

NCT05065840; October 4, 2021.

## Background

HPV-related anal cancer occurs in excess rates among people living with HIV (PLWH) [1], and has been increasing in incidence [1]. Notably, the incidence of anal cancer among men who have sex with men (MSM) is 20- to 40- fold greater relative to non-MSMs [2]. The Human Papillomavirus (HPV) is responsible for 90% of anal cancers where oncogenic HPV type 16 is responsible for 90% of anal cancers [3]. It is presumed the increased risk for anal cancer among PLWH is due to an impaired ability to clear HPV infections and increased reactivation of latent HPV infection. Of note, highly active antiretroviral therapy (HAART) has modest to no effect on HPV clearance or persistence; thus, other mechanisms may be involved that result in cellular immune dysfunction [4].

The safety and efficacy of the HPV vaccine has been evaluated in PLWH and is shown to be safe and highly immunogenic against oncogenic HPV types 16 and 18 [5–8]. The HPV vaccine also has been shown to decrease the risk of HPV-related anal intraepithelial neoplasia in a sample of MSMs [9]. Thus, anal cancer can be potentially a preventable disease through the use of the HPV vaccine [3]. However, very limited research has been conducted on the uptake of HPV vaccination among PLWH. One study found among a sample of young MSM’s who self-reported as HIV-positive, HPV vaccine initiation was 13.4% [10]. Although uptake is low, studies of the acceptability of the HPV vaccine has been found to be high among high risk groups like MSMs [11–13].

The United States’ Advisory Committee on Immunization Practices (ACIP) recommends vaccination up to age 26 years and recently FDA (Food and Drug Association) approved up to age 45 years for women and men [14]. ACIP also advises individuals who are immunocompromised to receive the 3-dose series of the HPV vaccine up to age 26 years of age and with shared clinical decision making for those 26 years and older. The Center for Disease Control and Prevention (CDC) urges catchup vaccination for adults who have not been previously vaccinated and remain vulnerable to develop preventable HPV-related cancers [15]. Yet, there is a dearth of studies that have tailored and implemented evidence-based approaches to promote HPV vaccination among PLWH and eligible for catchup vaccination. Since intervention development is costly, complex, and time consuming, we seek to refine and tailor an existing, evidence-based intervention and integrate in a new population and new setting. The CDC’s 4 Pillars™ Practice Transformation Program (4 Pillars™ Program) is a robust and empirically supported strategic approach that promotes the uptake of adult vaccinations and addresses facilitators and barriers at the patient, provider, and clinic level [16]. The 4 Pillars™ Program incorporates these recommendations via “a menu” of strategies to promote the establishment and maintenance of vaccination into routine practice (Table 1).

**Table 1:** The 4 Pillars Practice Transformation Program^TM^: Evidence-based strategies to increase vaccination

Pillar 1: Convenient and easy accessibility	• Use every patient visit type as an opportunity to vaccinate. • Offer open access/walk-in vaccination during office hours. • Promote simultaneous vaccination. • Hold express vaccination clinics outside normal office hours where only influenza or other adolescent vaccines are offered and systems for check-in, screening, and record-keeping are streamlined. • Create a dedicated vaccination station.
Pillar 2: Patient communication/education	• Provide information about vaccine preventable diseases at the beginning of every visit. • Enroll patients in electronic health portal. • Train staff to discuss vaccines during routine processes. • Discuss the serious nature of vaccine preventable diseases. • Use clinic messages, poster, fliers, electronic message board, website posting, and social media to promote vaccination. • Reach out by email, phone, text, mail, health portal to recommend vaccines that are due.
Pillar 3: Enhanced systems to promote vaccination	• Ensure sufficient vaccine inventory. • Assess vaccination eligibility for every patient encounter. • Assess immunizations as part of vital signs. • Review and update accurate EMR vaccination record keeping. • Establish standing order protocols.
Pillar 4: Motivation	• Create a chart to track progress. Set an improvement goal and regularly track progress. • Provide ongoing feedback to staff on vaccination progress. • Create a competitive challenge/provide reward for successful results among staff.

The 4 Pillars™ Program has shown to improve vaccination rates among high risk adults in primary care practices that successfully implemented strategies across the program [17, 18]. A randomized controlled cluster trial (RCCT) found the 4 Pillars Program significantly increased HPV vaccination among a cohort of 10,861 adolescent patients in primary care practices [19]. The intervention sites increased baseline HPV vaccination by 10.2 percentage points (PP) versus 7.3 PP in the control sites (*p* < .001) [19]. Furthermore, another large RCCT of adolescents found the 4 Pillars™ Program significantly increased baseline initiation of HPV vaccination by 17.1 PP (*p* < .001) and increased HPV completion by 14.8 PP (*p* < .001) [20]. These findings highlight the effectiveness of the 4 Pillars™ Program to increase HPV vaccination in the general population.

The Advancing HPV vaccination for HIV Positive Adults (CHAMPS) study seeks to expand the success of the 4 Pillars™ Program and tailor, refine, and implement in the HIV positive population, who are at high risk for HPV-related cancers and can obtain the most benefit from the vaccine. The strategies selected from the 4 Pillars™ Program are based on an extensive review of the HIV and related literature (Table 2) [21–28]. The intervention will be implemented in three HIV community clinics in Georgia, USA and enroll *n* = 365 PLWH, age 18–45 years, from those clinics. Guided by the RE-AIM framework, the proposed specific aims are:

1. Tailor and refine the 4 Pillars™ program for implementation in three HIV community clinics in Georgia.
2. Test the effectiveness of the 4 Pillars™ program as measured by an increase in uptake rate in initiation of the HPV vaccine (primary outcome) and vaccine completion (secondary outcome) compared to historical baseline vaccination rate (control) among PLWH. It is hypothesized after implementation of the 4 Pillars™ program, we estimate an uptake rate of > = 13.5% in initiation of HPV vaccination.
3. Identify mediators and potential moderators (HPV knowledge and awareness and vaccine hesitancy) of the intervention effects on HPV vaccination.
4. Assess the sustainability of the intervention in vaccine uptake post-intervention and assess scalability of the program for wider implementation via a future national randomized control trial.

**Table 2:** Overview of selected intervention strategies guided by the 4 Pillars™ Program

Pillar Strategy	Level	Intervention Component
Pillar 1: Convenience	Provider-	• Incorporate recommendation of the HPV vaccine with each clinic visit. • Perform HPV vaccination on-site
Pillar 2: Communication and education	Patient-	• Provide patient education on risk of HPV-related cancer and benefit of HPV vaccination
Pillar 3: Enhanced systems	Clinic-	• Provider and staff education on HPV vaccination via an in-service training • Document vaccination in EMR system
Pillar 4: Motivation	Clinic- Provider- Patient-	• Clinic designated Immunization Champion to provide coaching and motivation of regularly tracked vaccination progress • Provide patient check-in, reminders, and motivation for HPV vaccine completion • Communicate vaccination reminders by text, phone, and social media messaging

## Methods/design

**Fig. 1:**
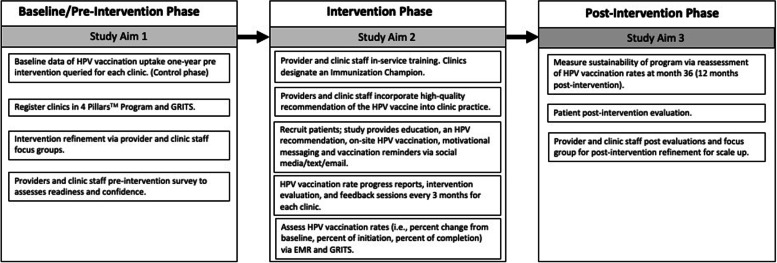
Schematic overview of the CHAMPS study

A pre-post intervention study design is used where HPV vaccination initiation and completion rates are measured before and after the intervention across the same clinics and enrolled participants (Fig. 1). HPV vaccination uptake 18 months before intervention will be queried from electronic medical records (EMR) and Georgia Registry of Immunization Transactions and Services (GRITS), which will serve as the historical control. GRITS is a population-based web application containing consolidated demographic and immunization history information. The use of a concurrent control is to reassess the background HPV uptake rate among PLWH during an adjacent time-period to post-intervention and similar population resources. The comparison of HPV vaccination rates pre- and post-intervention in the three selected representative HIV clinics in Georgia will be an exploratory goal of the trial due to the retrospective approach in the control phase and the prospective approach in the post intervention phase. A “within” analysis will be conducted to compare sites both pre- and post-intervention.

### Clinic selection and patient eligibility

Three HIV community clinics for this study were selected due to agreement to participate in the study, granted study access to electronic medical records, and willingness to make office changes to increase vaccination rates. Patients will be recruited from these three clinics and enrolled in the study based on the following eligibility criteria: 1) HIV positive; 2) 18–45 years of age; 3) understand English; 4) capable of informed consent; 5) have not received or completed the three dose HPV vaccine; 6) no contraindications to receiving the HPV vaccine (i.e., history of an anaphylactic allergy to latex, an immediate hypersensitivity to yeast, current moderate or severe acute illness, and/or are currently pregnant).

### Pre-implementation approach

During the pre-intervention phase of the trial, each clinic site enrolls patients who receive immunizations using the GRITS system. The GRITS system offers a variety of functions for health care providers including recording immunizations, validating immunization history, providing immunization recommendations, producing recall and reminder notices, generating vaccine usage and client reports, and performing data extraction. Clinics will register with the 4 Pillars™ Program and clinic staff will complete a pre-intervention survey that assesses readiness and confidence in increasing HPV vaccination and current vaccination practices. Providers and clinic staff will be asked to participate in a focus group for feedback on tailoring the intervention for their clinic population prior to program implementation.

### Clinic-level intervention approach

The 4 Pillars™ Program offers providers and clinic staff evidence-based strategies to increase HPV vaccination uptake via training and educational resources. This program will be refined to provide tailored training and motivation to HIV providers and clinic staff to recommend and administer the HPV vaccine to HIV patients at the infectious disease clinic. Providers and clinic staff who are interested in participating will “enroll” online and complete an electronic informed consent before participating in the focus groups and completing the evaluation surveys. Providers and clinic staff are offered an opportunity to attend an in-service training that will provide education, training, and resources to help increase HPV vaccination at their clinic. Participation in the in-service will be offered to the entire clinic with opportunity for continuing education (CE) units to be earned. The in-service component is delivered under the purpose of quality improvement and does not require informed consent to attend.

Components of the in-service training consist of education and resources related to the 4 Pillars™ program, epidemiology of HPV-related cancers among HIV positive individuals, ACIP’s guidelines for HPV vaccination for immunosuppressed patients, safety profile of the vaccine, and the importance and effectiveness of delivering evidence-based recommendations for HPV vaccination. Providers and clinic staff who are within scope of practice to administer the HPV vaccine are asked to recommend and administer the HPV vaccine to eligible patients during each routine clinic visit. Consenting providers and clinic staff are asked to complete pre-intervention evaluations, an intervention evaluation every 3 months, and post-intervention evaluations via a secured link to complete online. Alternatively, paper copies will be provided to the providers and clinic staff and administered by an Immunization Champion to those who choose not to access the evaluations by email.

Each clinic site identifies an Immunization Champion (a medical assistant, nurse, or clinic manager) who will work and motivate the clinic staff and participate in biweekly updates of progress with the research coordinator. The Immunization Champion (IC) will encourage, remind, and ensure timely documentation of the HPV vaccination within the clinic’s electronic medical records and within GRITS. The IC helps maintain stock and storage of the vaccine and identify and address any issues with the vaccine inventory. The IC assists patients to complete Merck’s Patient Assistance Program to cover vaccination for those who are uninsured and qualify for the program. Lastly, the IC will contact patients on the monthly call list to schedule appointments or assist in reminders to patients to schedule the next visit for the follow up HPV vaccine.

Clinics receive a progress report that documents the clinic’s vaccination progress every three months. The research coordinator schedules group feedback sessions via in-person or webinar with the IC every three months to discuss: 1) the intervention evaluations completed by the providers and staff; 2) to learn of any barriers to HPV vaccination at the clinic; 3) brainstorm strategies to overcome the barriers; and 4) quality assurance of intervention fidelity.

### Patient-level intervention approach

Eligible and consenting participants (*n* = 365) will be part of the intervention group and will receive recommendation for the HPV vaccine from providers and clinic staff. Enrolled participants will also complete a self-administered questionnaire at enrollment on a HIPPA compliant online data management database on a tablet device. The survey questions will include sociodemographic characteristics, knowledge of and attitudes towards HPV, HPV vaccination, and anal cancer, and vaccine hesitancy. Participants will be requested to provide consent for their HPV vaccination history to be verified with electronic EMR and GRITS. Participants will then watch a short video on HPV and HPV vaccination that can be viewed on their phones (or the study’s tablet device) while waiting to be seen. Potential participants will be asked to follow the study’s private Facebook page which offers additional educational information tailored towards individuals with HIV on HPV-related disease and general health promotion, and risk reduction tips. The Facebook page will also utilize Facebook Messenger (commonly known as Messenger). The proposed study will utilize Messenger to send reminders for follow up appointments for the next shot in the series and motivational messaging to encourage and promote receipt of the HPV vaccine. Participants will be contacted to complete a post-evaluation survey administered via online, telephone, or a mailed paper copy at 6–9 months after baseline. Participants will receive $25 incentive for completion of baseline and follow up study activities.

### Data analysis plan

#### Study outcomes

**Fig. 2:**
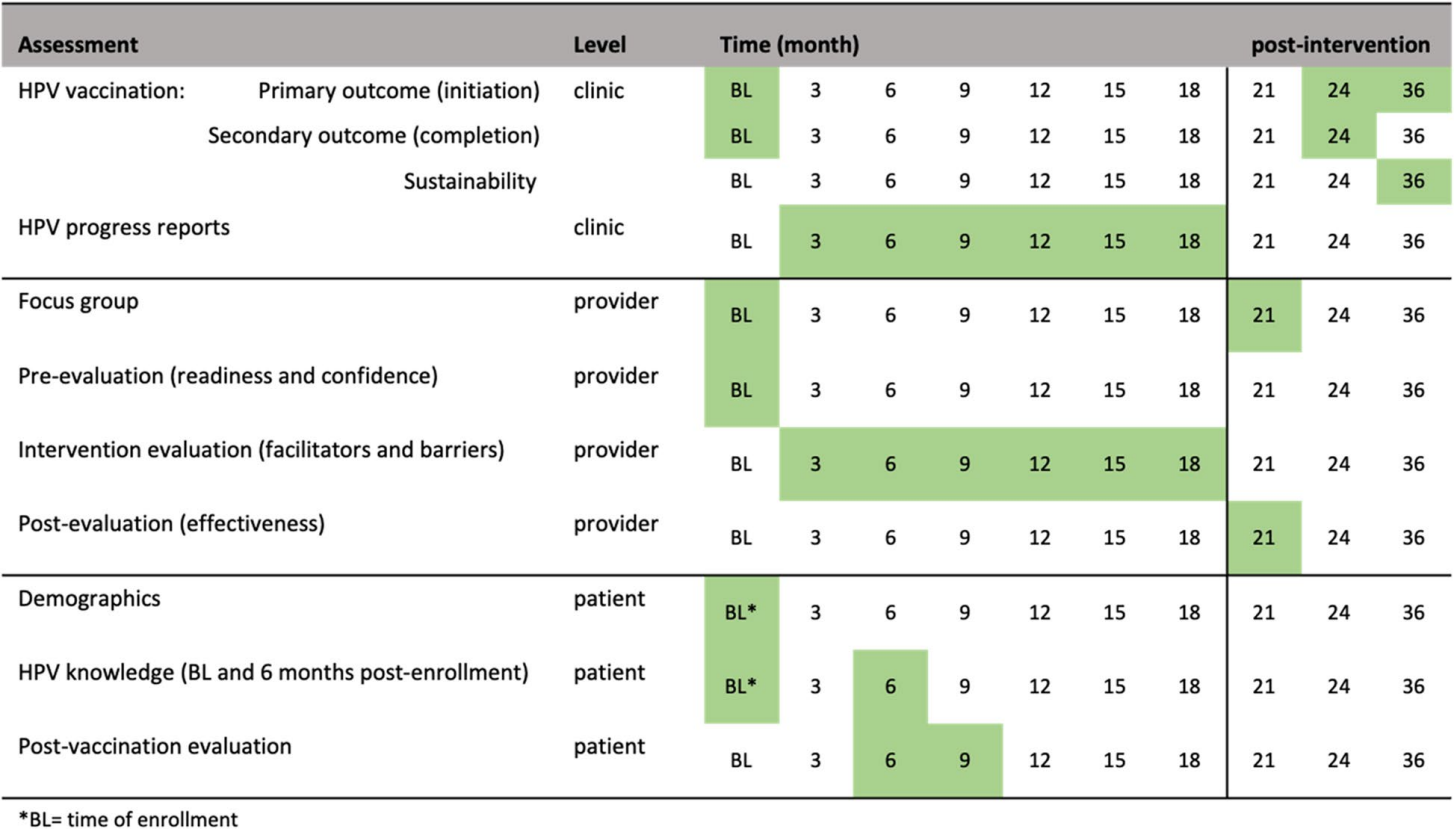
Summary of study assessments and time of collection

Initiation of the HPV vaccine is the primary outcome endpoint (Fig. 2). Initiation of the HPV vaccine is defined as receiving the first or second immunization from the series. This variable will be measured by electronic medical records and GRITS at baseline (historical control) and 24 months post baseline. We hypothesize the initiation rate will be higher than the historical control rate. Completion of the HPV vaccine is the secondary outcome variable. Completion is defined as receiving all three immunizations from the series, regardless of time. This variable will be measured by electronic medical records and GRITS baseline (control) and 24 months post baseline.

#### Process evaluation

The RE-AIM (Reach, Effectiveness/Efficacy, Adoption, Implementation, Maintenance) framework will guide planning, implementation, and evaluation of the 4 Pillars™ program. The study’s reach will be estimated from a quantitative perspective by estimating the target population that was exposed to the intervention. The clinic’s patient census data during the period of the implementation phase will be used to estimate the likely reach of the program across sites. Intervention effectiveness and efficacy will be measured by the change in uptake rate of vaccination (i.e., intervention effectiveness). We will calculate the percent change in initiation of the vaccine and percent change in completion of the vaccine from the control phase and 24 months post intervention, after adjusting for demographics differences (age, gender, race, healthcare insurance) in population. Providers and clinic staff will be asked to complete an evaluation of HPV vaccination progress every 3-months. We will also collect qualitative data from feedback sessions with the immunization champions to assess opportunities for and barriers to adoption. The post-evaluation interviews with providers and staff who implemented the program will assess extent of involvement, acceptance of the intervention, implementation fidelity, and extent of organizational spread of the intervention. We will assess the frequency, duration, and the extent to which the intervention was implemented as planned, participation attendance, and costs of implementation, as measured via the intervention and post-intervention evaluations. Intervention sustainability will be measured as the gains or maintenance of HPV vaccination rates post-delivery of the intervention. HPV vaccination rates will be calculated via EMR and GRITS at month 36 (12 months post intervention) and compare to HPV vaccination rates at month 24. Follow up assessments will measure penetration or the extent to which recommendation and administration of the HPV vaccination is integrated within the clinic.

#### Data analyses of primary outcomes

For analysis of the primary (HPV vaccination initiation) and secondary endpoints (HPV vaccination completion), the uptake rate of HPV vaccination pre- and post-intervention will be estimated separately with a 95% exact confidence interval using all eligible cases from both phases. The one-sample binomial exact test will be used to test whether the rate after the intervention is higher than the baseline rate (P0 = 13.5%). We will also perform the Chi-square test to compare the rate change between control and interventional phases, which will be exploratory. Logistic regression will be used to further adjust background difference in study population in pre- and post- intervention phases. For the longitudinal data collected from the intervention phase, the data structure holds multi-level information from patients, providers, and clinic levels. The goal of the analyses is to identify the factors that might impact HPV uptake from each level of information, which might lead to the future improvement of implementation strategy. Data will be described using summary statistics (e.g., quartiles, median, mean, standard deviation) for continuous variables and marginal distribution (frequency and percentage) for categorical variables. The univariate association with the HPV vaccination (yes vs. no) will be tested in logistic regression for each variable separately. The change of survey response between baseline and a follow-up time point will be tested by paired tests (e.g., paired t-test, McNemar test). Along with data visualization, all above mentioned descriptive and univariate association analyses will be repeated within each of the three clinics. Data will be pooled to build the multilevel analysis models, we mainly consider using the mixed-effect model and/or Bayesian multilevel modeling, in which the random effect will be considered at provider and clinical levels. We will follow the key modeling considerations listed by J.J. Hox [29] to identify the significant mediators and moderators at different levels that impact the uptake of HPV vaccination.

#### Secondary aims

To assess sustainability, HPV vaccination rates will be calculated at month 36 (12 months post-intervention). The change in HPV vaccination rates between month 24 and month 36 (12 months post-intervention) will be tested by McNemar test. The intervention will be deemed as sustaining its effect if rates of HPV vaccine initiation rates at month 36 remains or increases from month 24’s vaccination rates. To inform future scalability of the program, data from the evaluation and post-evaluation surveys will be described using summary statistics (e.g., quartiles, median, mean, standard deviation) for continuous variables and marginal distribution (frequency and percentage) for categorical variables. The similar analyses will be repeated within each of the three clinics. Additionally, we will conduct a post-intervention focus group consisting of the providers and clinic staff that participated in the program. The qualitative evaluation explore how implementation took place; the barriers to and facilitators of implementation success; ways to address any problems that may have occurred; and recommendations to refine the intervention for scale-up. The focus session will be recorded and transcribed where themes will be extracted and used for adaptation, scale-up considerations, and future research directions.

#### Statistical power

Based on our preliminary data, we found that HPV vaccination rate is around 13.5% (P0) in general, and another larger study in the literature found a very similar rate of 13.6% [10]. We powered the study to detect an uptake rate > 13.5% after the intervention. Thus, a sample size of 317 achieves 80% power to detect a superiority difference of 5% (PB-P0) using an exact one-sided test with a significance level (alpha) of 0.05. We anticipate a 5% superiority difference is reasonable to achieve with an uptake rate of 18.5% (PB) after the intervention. We will be able to reject the Null hypothesis and claim the uptake rate of > 13.5% after 54 patients have initiated the HPV test. After taking about 15% of the drop-off rate into account, we plan to include 365 participants among 3 clinics for the intervention phase. The calculation was by PASS 2020 for testing superiority of one proportion using the Exact test. The Null hypothesis is *P* < = 13.5% and the alternative hypothesis is *P* > 13.5%. For the control phase, we will query all eligible subjects from EMR database among the 3 clinics at 18 months pre-intervention, which could be approximately 2300 subjects [30]. Assuming we end up with the same number of subjects in both control (*N* = 317) and intervention phase (*N* = 317), we will have 81% statistical power to detect an HPV uptake rate difference of 9% (22.5% vs. 13.5%) by two-sided Fishers’ Exact Test and under significance level of 0.05. We anticipate such a difference would be feasible based on our best knowledge and the literature. Regarding the patient- level component of the intervention, all incoming eligible patients will be influenced, and hence 365 participants are the minimum number to capture the follow-up information, the actual number of sample size used for the calculation of HPV vaccination rate will be larger as the vaccination status will be captured automatically in EMR without a consent.

## Discussion

The underutilization of HPV vaccination is a national problem that has been identified by the President’s Cancer Panel as a serious but correctable threat to the progress against cancer [31]. However, few studies have focused on the high-risk HIV population—an aging population that is increasingly managing other co-morbidities with their HIV diagnoses, including cancer. HPV vaccination is a form of primary cancer prevention that is imperative for a successful cancer control plan that may reduce the untimely death and clinical burden of HIV patients from several potentially vaccine-preventable HPV-related cancers including anal, cervical, vulvar, vaginal, penile, and oropharyngeal cancers. With an aging HIV population, it is an essential public health goal to provide the necessary resources and cancer prevention strategies for PLWH to achieve a normal life expectancy and quality of life. The CHAMPS study is the next step to achieving this goal for high-risk HIV-positive populations.

### Trial registration

NCT05065840; Registered on October 4, 2021.

## Data Availability

The datasets used and/or analyzed during the current study will be available from the corresponding author on reasonable request.

## Abbreviations

HPV: Human Papillomavirus
HIV/AIDS: Human Immunodeficiency virus/ Acquired immunodeficiency syndrome
PLWH: People living with HIV
MSM: Men who have sex with men
HAART: Highly active antiretroviral therapy
FDA: Food and Drug Administration
CDC: Centers for disease control and prevention
PP: Percentage points
CHAMPS: Advancing HPV vaccination in HIV positive adults
RE-AIM: Reach, Effectiveness/Efficacy, Adoption, Implementation, Maintenance
EMR: Electronic medical records
GRITS: Georgia Registry of Immunization Transactions and Services
CE: Continuing education
IC: Immunization champion
PASS: Power analysis & Sample size
